# Determinants of food insecurity in homeless people: evidence from the territory-wide homeless census in Hong Kong

**DOI:** 10.1017/S1368980025100980

**Published:** 2025-08-27

**Authors:** Gary Ka-Ki Chung, Crystal Ying Chan, Siu Ming Chan, Hung Wong

**Affiliations:** 1 JC School of Public Health and Primary Care, Faculty of Medicine, The Chinese University of Hong Kong, Hong Kong, China; 2 CUHK Institute of Health Equity, The Chinese University of Hong Kong, Hong Kong, China; 3 Department of Social and Behavioural Sciences, The City University of Hong Kong, Hong Kong, China; 4 Department of Social Work, The Chinese University of Hong Kong, Hong Kong, China

**Keywords:** Food insecurity, Homelessness, Determinants, Hong Kong

## Abstract

**Objective:**

With the re-emerging homelessness issue in Hong Kong, given its least affordable housing, securing food to meet the basic physiological needs is of priority concern for the homeless. This study aims to examine the situation and determinants of food insecurity among the homeless in Hong Kong.

**Design:**

Cross-sectional survey on food insecurity level, socio-demographic characteristics, homeless experiences and health status and behaviours.

**Setting:**

360 community hot spots of homeless people identified by NGO and experienced social workers in different districts of Hong Kong in 2021.

**Participants:**

711 individuals experiencing homelessness.

**Results:**

The observed prevalence of low, medium and high food insecurity levels was 37·4 %, 20·4 % and 42·2 %, respectively. Results from multivariable ordinal logistic regression showed that older, female, non-Chinese and non-married respondents were inversely associated with food insecurity, whereas having sufficient savings for more proper housing was the primary determinant among socio-economic indicators. In addition to reasons for homelessness, risk factors of food insecurity included living in guesthouses/hotels and difficulties due to government measures on homeless control. Except for disability, both self-rated physical and mental health statuses showed dose–response relationships with food insecurity level.

**Conclusions:**

The substantial individual variations in socio-demographic statuses, homeless experiences and health deficits shaped the differential risks of food insecurity within the homeless community in Hong Kong. Targeted homeless programmes should go beyond the conventionally vulnerable groups but consider the multifaceted nature of homeless experiences in relation to food access and integrate health assessments to holistically support the homeless.

Housing unaffordability has been a persistent and complex issue in Hong Kong, posing significant social and public health challenges in the community^(1,2)^. Despite recent slight improvement due to policy attention from both the Hong Kong and China’s central governments, Hong Kong continues to have the least affordable housing across the globe. Notably, the Demographia International Housing Affordability report revealed that Hong Kong’s housing affordability ratio reached a staggering 20·8 in 2019 with the median housing cost in general exceeding 15 times the annual median pre-tax household income over the past decade, significantly worse than other advanced economies such as Sydney and Vancouver^(3)^. In addition, the public housing system in Hong Kong is heavily burdened, resulting in a persistently long average waiting time for public rental housing of at least 5 years^(4)^. As a result of the enduring housing unaffordability and the heavily burdened public housing system, homelessness has resurfaced as a prominent issue in Hong Kong. Specifically, the number of street sleepers (i.e. those sleeping in parks, sportsground, pavements, footbridges, subways or other public external places), which had reduced to 327 individuals by 2007, gradually rebounded to 1580 in 2020/21 and has remained high in recent years^(5,6)^. The scale of homelessness is indeed greater when considering those living in temporary shelters, short-term hostels or social housing and guesthouses, who are also regarded as homeless in a boarder sense. Moreover, the situation further deteriorated during the COVID-19 pandemic due to economic recession and the stringent containment measures such as the suspension of 24-hour restaurants, stores and public facilities, pushing the socially vulnerable to become homeless and resort to rough sleeping or relocation^(6–8)^.

Among the various health and social needs of the homeless, securing food to meet the basic physiological needs for survival is of priority concern^(9)^. Existing research highlights the major barriers to achieving food security for homeless individuals. For instance, homeless individuals often face challenges in maintaining regular meal timing due to unstable living situations and limited access to cooking and storage resources in shelters or on the street^(10,11)^. The lack of control over healthy food choices is also common, given the usually higher cost of nutritious foods, leading to dietary inadequacies even in core food groups such as cereals and vegetables^(11–13)^. These barriers to ensuring food access have significant public health implications, as homeless individuals experiencing food insecurity are closely linked to poorer health outcomes and greater healthcare needs^(14–16)^. While food assistance programmes may provide temporary relief for food deprivation among the homeless population living in commonly recognised hotspots or shelters, these support programmes are usually deemed inadequate to improve overall food security due to limited capacity, insufficient provision of nutrient-dense foods, irregular availability of services, as well as ineffective communication and information dissemination^(17)^. Also, these programmes may not reach transients without a fixed residence or those who are relatively hidden in areas not typically associated with homelessness.

From the adaptation perspective, the levelling and differentiation hypotheses have been proposed to understand the relationship between homelessness and food insecurity^(18)^. The levelling hypothesis suggests the shared experience and barriers of homelessness are sufficiently overwhelming stressors that any individual variations within the homeless population could hardly determine their vulnerability to hunger, whereas the differentiation hypothesis acknowledges that individual characteristics and circumstances contribute to varying levels of resources and obstacles, including individual socio-demographic statuses, homeless experiences and support network and individual deficits, that differentiate their ability to adapt to food insecurity^(18)^. If the differentiation hypothesis holds true, identifying the major predictors of food insecurity would become crucial to inform possible entry points of interventions in the community. While studies on the interplay between these hunger-reducing resources and hunger-elevating obstacles in the homeless are relatively abundant in developed Western settings^(19–22)^, no relevant evidence is available in Hong Kong for comparisons. Therefore, this study aims to test the levelling and differentiation hypotheses by assessing whether, or to what extent, individual variations in the above-mentioned domains of resources and obstacles could determine the risk of food insecurity in the homeless population under the context of Hong Kong.

## Methodology

### Study population

This study utilised survey data from the ‘Hong Kong Homeless Census 2021’ project, which examined the homelessness situation and characteristics of homeless people in Hong Kong^(23)^. Conducted by academic institutions and non-governmental organisations (NGO) in 2021, this study stands as the largest territory-wide survey of the homeless population in Hong Kong. Eligibility criteria for participation involved individuals experiencing homelessness, including not only those sleeping rough on the streets but also those residing in temporary shelters, short-term hostels, guesthouses or hotels. To be included in this Census, individuals had to be present at pre-determined locations on the census night and identified by service organisations as homeless, indicating their accommodation status resulting from homelessness.

The scope of this Census covered more than 360 hot spots of homeless people, as identified by NGO and experienced social workers, in different districts of Hong Kong. The data were collected from the evening (i.e. 19.00) of 9 July 2021 to the small hours (i.e. 03.00) of 10 July 2021. Over 300 volunteers from NGO, universities and tertiary institutions, who underwent training sessions co-organised by universities and NGO, served as interviewers. Each team, comprising 2–3 interviewers and 1–2 team leaders, was assigned to one route and invited the homeless people they met along the designated route to complete a questionnaire. Coordination between team leaders ensured accurate recording in overlapping zones. To maximise the reach to homeless individuals, each team would revisit the same location thrice if empty beds were found without occupants.

In total, 1532 homeless people were identified during the survey period. Trained interviewers engaged with 1103 homeless individuals and successfully collected 719 questionnaires, with a response rate of 65·2 %, as the remaining 384 approached individuals refused to participate into this study due to various potential reasons including suspicions and a lack of rapport, sensitivity of topics (e.g. substance use), severe mental health issues, language barriers and perceived time burden for participation. Considering the response quality, 711 questionnaires were deemed valid for data analysis. Written informed consent was obtained from each participant after clear explanations on the purpose, procedures and objectives of the study in clear and accessible language, as well as assessments on participants’ capacity to provide consent, by our interviewers who underwent ethical research and sensitivity training. Ethical standards, including informed consent, anonymisation and data confidentiality, were ensured during the data collection process by all involved organisations, in adherence to established ethical guidelines.

### Measurements

#### Food insecurity level

Two questions from the Food Insecurity Experience Scale^(24)^ were used to examine the experience of food insecurity: (i) Was there a time when you were worried you would not have enough food to eat because of a lack of money or other resources? and (ii) Was there a time when you had to skip a meal because there was not enough money or other resources to get food during the past 12 months? Respondents screened positive in none, either one and both items were classified into ‘low’, ‘medium’ and ‘high’ level of food insecurity, respectively.

#### Predictors of food insecurity level

To test the levelling and differentiation hypotheses, multiple aspects of hunger-reducing resources and hunger-elevating obstacles in the homeless were measured as follows:

##### Demographic and socio-economic statuses

The questionnaire collected demographic characteristics of homeless people, including age, gender (i.e. male and female), ethnicity (i.e. Chinese and non-Chinese), marital status (i.e. currently married and non-married, which includes the single, widowed, separated and divorced). As for socio-economic statuses, data on educational attainment (i.e. primary or below, secondary and tertiary or above), current employment status (i.e. employed and unemployed) and the status of means-tested Comprehensive Social Security Assistance (CSSA) were obtained.

##### Homeless experiences and support network

Location of residence was recorded when trained interviewers identified homeless individuals on the spot, which was classified into six groups including (i) parks/sportsgrounds/car parks, (ii) pavements/footbridges/subways, (iii) other public external places, (iv) hostels/social housing, (v) guesthouses/hotels and (vi) temporary shelters. In addition, respondents were asked about their total duration of homelessness and whether they had enough savings for more proper housing. Also, reasons for homelessness were collected and re-grouped into four main categories, including (i) housing-related, (ii) economic, (iii) personal and (iv) family reasons. Specifically, examples of housing-related reasons included unaffordable rent, eviction by the landlord, demolition of previous accommodation, poor living conditions or flea infestations, while economic reasons covered unemployment and money saving. Personal reasons included personal choices, health reasons and being discharged from hospital, prison or drug treatment centre, whereas family reasons were related to divorce and poor relationships with family members or previous tenants. Regarding social ties of the homeless, respondents were asked whether they had regular contact with family and friends and whether they had contacted social workers or social service agencies. As for experiences of homeless control measures, respondents were asked whether they had faced difficulties due to five common government measures, including (i) personal belongings being tossed away, (ii) being dispersed or evicted, (iii) sleeping locations being blocked off by the government, (iv) sleeping locations being closed due to COVID-19 lockdown and (v) being unable to sleep rough because of floor washing or spilling of odorous powders. Respondents who reported sometimes or usually experiencing any of these five measures were deemed to be facing difficulties due to government measures.

##### Individual health deficits

Respondents were asked to self-report whether they engaged in risky behaviours including drug abuse, alcohol abuse and gambling. In addition, both physical and mental health status of homeless individuals were assessed. Respondents were asked about the presence of physical disability or chronic diseases that require regular follow-ups, and to self-rate their physical health status with five options ranging from poor to excellent^(25)^. Mental health status was assessed by the 4-item Patient Health Questionnaire, with four options ranging from not at all to nearly every day^(26,27)^. Respondents were classified into normal (0–2), mild (3–5), moderate (6–8) and severe (9–12) based on their 4-item Patient Health Questionnaire score.

### Statistical analysis

Descriptive statistics of the sample characteristics, both overall and stratified by food insecurity levels, are presented as mean with sd for continuous variables and frequencies with percentages for categorical variables. As food insecurity status was categorised into three levels, univariate and multivariable ordinal logistic regression analyses were employed to examine the associations of demographic and socio-economic statuses, homeless experiences and individual health deficits with the risk of food insecurity. The proportional odds assumption was checked with a global *P*-value > 0·05 in the final model, indicating no apparent violation of the assumption in the analyses. Due to a substantial number of missing values in the dataset under the constrained setting for data collection, multiple imputation by chained equations was adopted in the multivariable analyses to estimate a set of plausible values for the missing data based on the distribution of the observed data^(28)^. The statistical package Stata version 17 was employed. All statistical tests were two-tailed with a significance level of *P*-value < 0·05.

## Results

### Basic sample characteristics

Descriptive statistics of the overall sample and stratified sample by food insecurity level are presented in Table 1. Among the sampled homeless respondents, more than half faced high or medium food insecurity (42·2 % and 20·4 %, respectively). The mean age was 56·8 (sd 11·9) year, with low proportions of female (16·1 %) and non-Chinese (8·7 %) but high proportion of non-married respondents (82·4 %). As for socio-economic indicators, 35·1 % respondents attained only primary education or below and 8·3 % attained tertiary education, whereas 31·1 % were employed, and 41·6 % were on CSSA. In terms of homeless experience, over half of respondents were homeless for more than 1 year in total (60·5 %), but there was also a substantial proportion who became homeless for less than 3 months (23·6 %). Over half respondents were living in roofless places including parks/sportsgrounds/car parks (24·2 %), pavements/footbridges/subways (15·0 %) and other external places (18·1 %), whereas the rest were living in roofed place such as hostels/social housing (17·8 %), guesthouses/hotels (18·6 %) and temporary shelters (6·2 %) at the time of interview. About one-fifth stayed homeless despite having enough savings for proper housing (19·1 %). The proportions of respondents who reported housing-related, economic, personal and family issues as the reasons for homelessness were 55·8 %, 31·2 %, 21·1 % and 18·4 %, respectively. Most respondents had contact with families and friends (52·2 %) as well as with NGO and social workers (73·2 %), whereas about half had ever faced difficulties due to government measures (46·6 %). As for health and social behaviours, the corresponding proportions of drug abuse, alcohol abuse and gambling were 8·2 %, 19·4 % and 17·7 %, and that of respondents who had chronic diseases and disability were 39·0 % and 15·5 %, respectively. About half of respondents rated their health as either poor (15·2 %) or fair (34·8 %) and showed at least mild mental health symptoms (46·6 % in total). Further details of sample characteristics by food insecurity level are displayed in Table 1.

**Table 1.** Basic characteristics of the sampled homeless individuals in Hong Kong

	Overall sample (*n* 711)		Food insecurity status*					
				Low food insecurity		Medium food insecurity		High food insecurity	
				(*n* 249; 37·4 %)		(*n* 136; 20·4 %)		(*n* 281; 42·2 %)	
	*n* or Mean	% or sd	*n* or Mean	% or sd	*n* or Mean	% or sd	*n* or Mean	% or sd
Socio-demographic factors								
Age^†^	56·8	11·9	58·5	11·7	57·6	12·9	54·3	11·2
Gender, % female^‡^	114	16·1	39	15·7	24	17·8	42	14·9
Ethnicity, % non-Chinese^§^	61	8·7	22	8·9	15	11·0	24	8·7
Marital status, % non-married^||^	554	82·4	193	82·1	113	85·6	220	80·6
Educational attainment^¶^								
Primary or below	236	35·1	82	33·7	56	42·4	81	30·2
Secondary	380	56·5	140	57·6	66	50·0	165	61·6
Tertiary or above	56	8·3	21	8·6	10	7·6	22	8·2
Current employment status, % employed**	219	31·1	88	35·6	36	26·5	89	31·8
CSSA status, % on CSSA	296	41·6	96	38·6	62	45·6	118	42·0
Homeless experience								
Homeless duration^††^								
Less than 3 months	157	23·6	54	22·9	26	20·3	66	25·6
3–6 months	48	7·2	14	5·9	8	6·3	23	8·9
7–12 months	58	8·7	20	8·5	13	10·2	24	9·3
13–24 months	100	15·0	34	14·4	19	14·8	42	16·3
25–60 months	151	22·7	58	24·6	30	23·4	55	21·3
More than 60 months	152	22·8	56	23·7	32	25·0	48	18·6
Homeless location^‡‡^								
Parks/sportsgrounds/car parks	171	24·2	57	23·0	39	28·7	56	20·1
Pavements/footbridges/subways	106	15·0	47	19·0	15	11·0	30	10·8
Other public external places	128	18·1	47	19·0	25	18·4	50	18·0
Hostels/social housing	126	17·8	43	17·3	25	18·4	57	20·5
Guesthouses/hotels	131	18·6	34	13·7	24	17·6	69	24·8
Temporary shelters	44	6·2	20	8·1	8	5·9	16	5·8
Enough savings for proper housing^§§^	131	19·1	74	30·8	13	9·9	42	15·0
Housing-related reasons for homelessness	397	55·8	126	50·6	76	55·9	168	59·8
Economic reasons for homelessness	222	31·2	73	29·3	36	26·5	105	37·4
Personal reasons for homelessness	150	21·1	68	27·3	26	19·1	49	17·4
Family reasons for homelessness	131	18·4	51	20·5	26	19·1	50	17·8
Having contact with families and friends^||||^	364	52·2	140	57·1	69	50·7	139	49·8
Having contact with NGO and social workers^¶¶^	507	73·2	176	72·1	102	75·0	210	75·3
Ever faced difficulties due to government measures***	290	46·6	83	37·7	63	50·0	130	51·8
Health and social behaviours								
Drug abuse^†††^	56	8·2	18	7·4	9	6·7	26	9·4
Alcohol abuse^‡‡‡^	135	19·4	44	18·0	29	21·3	54	19·4
Gambling^§§§^	123	17·7	52	21·2	25	18·4	40	14·2
Chronic disease^||||||^	272	39·0	83	33·6	49	36·0	130	46·4
Disability^¶¶¶^	108	15·5	40	16·2	18	13·2	43	15·4
Self-rated health status****								
Excellent	61	8·8	34	13·8	11	8·2	13	4·7
Very good	148	21·4	58	23·6	33	24·6	47	16·8
Good	138	19·9	61	24·8	21	15·7	53	19·0
Fair	241	34·8	70	28·5	47	35·1	109	39·1
Poor	105	15·2	23	9·3	22	16·4	57	20·4
Mental health status^††††^								
Normal	356	53·4	169	71·0	83	62·4	92	33·6
Mild	147	22·0	36	15·1	17	12·8	88	32·1
Moderate	73	10·9	15	6·3	19	14·3	36	13·1
Severe	91	13·6	18	7·6	14	10·5	58	21·2

Missing: *45; ^†^18; ^‡^3; ^§^9; ^||^39; ^¶^39; **6; ^††^45; ^‡‡^5; ^§§^25; ^||||^14; ^¶¶^18; ***89; ^†††^26; ^‡‡‡^16; ^§§§^15; ^||||||^14; ^¶¶¶^14; ****48; ^††††^44.

### Determinants of food insecurity level

Results from ordinal logistic regression analyses are presented in Table 2. In the adjusted model, demographic factors including older age (aOR = 0·97 (95 % CI: 0·96, 0·99)), female gender (aOR = 0·51 (0·32, 0·80)), being non-Chinese (aOR = 0·44 (0·24, 0·81)) and being non-married (aOR = 0·55 (0·35, 0·86)) were protective factors against food insecurity, although educational attainment, current employment status and CSSA status did not predict food insecurity. Regarding homeless experience, respondents living in guesthouses/hotels tended to experience greater food insecurity (aOR = 1·98 (1·08, 3·62), compared with those sleeping in parks/sportsgrounds/car parks), whereas having sufficient savings for more proper housing was associated with lower food insecurity (aOR = 0·48 (0·32, 0·74)). In addition, lower food insecurity was observed in respondents who reported personal issues as the reason for homelessness (aOR = 0·54 (0·36, 0·79)), while greater food insecurity was found in those who faced difficulties due to government measures (aOR = 1·81 (1·29, 2·55)). Nonetheless, total homeless duration, other reasons for homelessness and social support from family, friends, NGO or social workers did not predict food insecurity. As for health and social behaviours, disability was associated with lower food insecurity (aOR = 0·51 (0·32, 0·83)), whereas greater food insecurity level was observed in respondents with poorer self-rated health (poor: aOR = 2·96 (1·32, 6·63); fair: aOR = 2·42 (1·23, 4·77); good: aOR = 1·68 (0·84, 3·34); very good: aOR = 2·08 (1·07, 4·05), compared with excellent health) and mental health status (severe: aOR = 4·46 (2·61, 7·61); moderate: aOR = 2·35 (1·40, 3·95); mild: aOR = 3·34 (2·14, 5·19), compared with normal mental health). However, risky behaviours including drug use, alcohol abuse and gambling were not statistically significant predictors of food insecurity.

**Table 2.** Associations of socio-demographic factors, homeless experience and health and social behaviours with food insecurity level based on multivariable ordinal logistic regression analysis

	Unadjusted model			Adjusted model		
	OR	95 % CI	*P*-value	aOR	95 % CI	*P*-value
Socio-demographic factors						
Age	0·98	0·96, 0·99	< 0·001	0·97	0·96, 0·99	0·001
Female gender	0·91	0·63, 1·32	0·626	0·51	0·32, 0·80	0·003
Non-Chinese	0·95	0·58, 1·54	0·829	0·44	0·24, 0·81	0·008
Non-married	0·87	0·59, 1·26	0·450	0·55	0·35, 0·86	0·009
Educational attainment						
Primary or below	ref			ref		
Secondary	1·15	0·84, 1·57	0·393	0·89	0·61, 1·30	0·544
Tertiary or above	1·01	0·58, 1·77	0·959	1·01	0·53, 1·96	0·966
Currently employed	0·89	0·65, 1·20	0·440	0·83	0·56, 1·22	0·340
On CSSA	1·15	0·87, 1·53	0·323	1·13	0·78, 1·64	0·509
Homeless experience						
Homeless duration						
Less than 3 months	ref			ref		
3–6 months	1·25	0·68, 2·30	0·473	1·34	0·67, 2·68	0·415
7–12 months	0·90	0·51, 1·57	0·708	1·10	0·57, 2·11	0·775
13–24 months	0·98	0·61, 1·56	0·918	1·33	0·76, 2·33	0·310
25–60 months	0·76	0·51, 1·15	0·195	1·02	0·61, 1·70	0·947
More than 60 months	0·65	0·44, 0·98	0·040	0·76	0·46, 1·27	0·302
Homeless location						
Parks/sportsgrounds/car parks	ref			ref		
Pavements/footbridges/subways	0·65	0·40, 1·06	0·088	0·84	0·47, 1·53	0·573
Other public external places	1·02	0·65, 1·62	0·916	1·10	0·63, 1·93	0·731
Hostels/social housing	1·24	0·79, 1·94	0·351	1·64	0·87, 3·12	0·127
Guesthouses/hotels	1·70	1·09, 2·64	0·020	1·98	1·08, 3·62	0·027
Temporary shelters	0·80	0·42, 1·51	0·485	0·84	0·40, 1·75	0·639
Enough savings for proper housing	0·42	0·29, 0·61	< 0·001	0·48	0·32, 0·74	0·001
Housing-related reasons for homelessness	1·36	1·03, 1·80	0·031	1·20	0·86, 1·68	0·285
Economic reasons for homelessness	1·42	1·05, 1·92	0·023	1·10	0·78, 1·56	0·594
Personal reasons for homelessness	0·64	0·46, 0·91	0·012	0·54	0·36, 0·79	0·002
Family reasons for homelessness	0·88	0·61, 1·25	0·466	0·88	0·58, 1·33	0·535
Having contact with families and friends	0·78	0·59, 1·03	0·080	0·82	0·59, 1·13	0·225
Having contact with NGO and social workers	1·12	0·80, 1·56	0·503	0·83	0·55, 1·25	0·366
Ever faced difficulties due to government measures	1·62	1·22, 2·16	0·001	1·81	1·29, 2·55	0·001
Health and social behaviours						
Drug abuse	1·32	0·79, 2·22	0·294	1·16	0·63, 2·16	0·631
Alcohol abuse	1·04	0·73, 1·48	0·812	1·09	0·72, 1·65	0·693
Gambling	0·68	0·47, 0·97	0·035	0·65	0·42, 1·03	0·065
Chronic disease	1·60	1·20, 2·14	0·002	1·30	0·88, 1·91	0·189
Disability	0·94	0·64, 1·39	0·765	0·51	0·32, 0·83	0·006
Self-rated health status						
Excellent	ref			ref		
Very good	1·81	1·00, 3·28	0·051	2·08	1·07, 4·05	0·031
Good	1·85	1·01, 3·39	0·045	1·68	0·84, 3·34	0·142
Fair	2·95	1·68, 5·18	< 0·001	2·42	1·23, 4·77	0·011
Poor	4·24	2·26, 7·98	< 0·001	2·96	1·32, 6·63	0·009
Mental health status						
Normal	ref			ref		
Mild	3·79	2·56, 5·61	< 0·001	3·34	2·14, 5·19	< 0·001
Moderate	2·93	1·83, 4·68	< 0·001	2·35	1·40, 3·95	0·001
Severe	4·54	2·86, 7·20	< 0·001	4·46	2·61, 7·61	< 0·001

## Discussion

### Summary of findings

As the first study on the determinants of food insecurity of the homeless in Hong Kong, our findings lent more support for the differentiation hypothesis over the levelling hypothesis that responses to homelessness in relation to food access are shaped by variations in the balance between hunger-reducing resources and hunger-elevating obstacles, including demographic and socio-economic statuses, experiences and reasons of homelessness, as well as individual health deficits, that in turn structure the adaptation process in the homeless. Specifically, homeless people who were younger, male, Chinese and ever-married were at higher risk of food insecurity. Monetary resources in terms of savings, rather than education and employment status, served as the major protective socio-economic determinant of food insecurity. Living locations and reasons of homelessness also played a role, in which the risk of food insecurity was higher in those living in guesthouses or hotels compared with street sleepers and lower in those becoming homeless due to personal affairs. In addition, homeless people who faced difficulties due to government measures, such as eviction, were at elevated risk of food insecurity. Regarding individual health deficits, except for a lower risk in the disabled, greater food insecurity was observed in those with poorer self-rated health status and mental health status. Due to the lack of existing local evidence for comparisons, plausible speculations are discussed below to allow a better understanding of our findings and their implications in the context of Hong Kong.

### Interpretation of findings

Contrary to common assumptions, our findings suggested that older adults, women, ethnic minorities and non-married individuals, who are often considered more vulnerable in many aspects of life, were associated with a lower risk of food insecurity within the context of homelessness. Such reversed associations may be the result of social welfare and interventions targeted at these vulnerable groups, especially for older adults who are entitled to additional allowances (e.g. old age allowance, old age living allowance etc.) and social services including food aids and hot meal support initiated by local church groups and NGO. Nonetheless, gender norms and disparities in help-seeking beliefs could have also played a part among the disadvantaged. Earlier research reported a stronger association of homelessness with food insecurity in men than in women^(29)^ and that homeless men tended to engage in more fasting practices and have poorer diet quality, which may be largely attributable to their lower willingness to receive services and informal social support^(18)^. In addition, a recent local study suggested the less positive help-seeking attitudes in local Chinese compared to Western people in Hong Kong^(30)^. Although this study did not specifically focus on food support for the homeless, it provided clues that local Chinese may be more reluctant to access services even when they are in need. Moreover, the higher risk of food insecurity in the married may be related to their caregiving responsibilities and potential self-sacrificing behaviours by prioritising the needs of family members over themselves^(10,31)^. Further homeless research, in particular qualitative interviews with these social groups, is warranted to delineate the mechanisms and dynamics between these demographic statuses and social support systems in relation to their better (or improved) food access within the homeless community.

Regarding socio-economic statuses, our findings indicated that among the homeless, savings play a crucial role in mitigating food insecurity. The availability of readily accessible and disposable money in the form of savings provides a tangible socio-economic resource that directly helps buffer against financial shocks under economic recession and secure basic physiological needs such as access to food for the homeless^(32,33)^. Nonetheless, while the relatively upstream socio-economic statuses, such as educational attainment and employment status, are important in preventing homelessness^(34–36)^ and ill-health^(37–39)^ in the general population, their influence on addressing food insecurity appears to be less pronounced compared with monetary resources once individuals have already become homeless. The effect of sufficient savings also remained strong after considering the CSSA status, which typically involves a food subsidy, accentuating the pivotal role of wealth accumulation in this population. Our result implies that homelessness *per se* may be sufficiently handicapping to create significant socio-economic challenges that overshadow the distal beneficial effects of education and employment or short-term impact of financial subsidies, making personal assets, such as savings, the primary socio-economic determinant of food insecurity within the homeless population. To facilitate a more effective saving practice, programmes on promoting financial literacy and addressing the potential constraints to saving may be crucial to empower the homeless for better financial decision-making and enhance their resistance to food insecurity or other health shocks^(40)^.

In addition to demographic and socio-economic statuses, homeless reasons and experiences also determined the risk of food insecurity. People becoming homeless due to personal reasons tended to be less food insecure than those reporting no personal affairs, but a mix of other reasons related to housing, economic conditions and family issues. However, the independent effects of each of these non-personal reasons were NS. Such findings reiterated the socio-ecological and intersectional nature of these precarities in shaping disparities in food access at inter-personal and community levels above and beyond individual factors^(41)^. As for homeless status, while the generally lower risk of food insecurity in street sleepers was revealed by our previous work^(23)^, we further examined such phenomenon with a finer classification of their living locations. There were negligible differences in the risk of food insecurity across street sleepers living in different public external locations; nonetheless, such risk was particularly higher in homeless guesthouse or hotel renters compared with street sleepers and those living in temporary shelters. Although a relatively better financial condition may be expected in the homeless who manage to cover the significant accommodation fees, it may also reflect a lower disposable income for food purchases as a trade-off between living environment and diet quality, given the limited resources^(42–44)^. In addition, inadequate access to food support programmes may contribute to the higher risk in those living in guesthouses or hotels. In Hong Kong, several charity parties and NGO regularly provide free meal boxes or other food items at well-known public spots or clusters of homeless people^(45)^. Nonetheless, as the homeless guesthouse or hotel renters are scattered widely across the territory and food inaccessibility is rarely deemed problematic in these settings, food donations may become less accessible unless they actively seek support somewhere else. Similar logic can be applied to explain the observed adverse impact on food insecurity due to government measures on homeless control and eviction. When homeless individuals are evicted, they become highly mobile and less accessible, and any established support networks may no longer function effectively in a new setting^(46,47)^. To better address food insecurity in the homeless, policymakers should shift the focus from eviction to support measures that accommodate the homeless in temporary transitional shelters and offer tailored services to address their social and health needs. At the community level, enhancing the communication network with the homeless and the transparency of information related to food support programmes may facilitate food access for these highly invisible groups of homeless people.

Last, largely consistent with the existing literature^(16,20,48)^, individual health deficits in terms of self-rated physical and mental health were strongly associated with food insecurity in the homeless. The exceptional lower risk of food insecurity in homeless individuals living with disability was perhaps due to additional disability allowances and social service provision to support the disabled. The associations between health and food insecurity in the homeless appear to be bilateral. Previous studies demonstrated that the associations with both physical and mental health symptoms were plausibly attributable to the lower emotional distress tolerance and sense of control in the food-insecure individuals^(49,50)^. Other research also showed a common and persistent food insecurity issue in homeless individuals with mental illness^(51)^, whereas the presence of physical and mental health issues would in turn heighten the risk of food insecurity^(51,52)^. Regardless of the directionality of such an association, our findings underscore the importance of integrating health screenings into support programmes designed to reduce housing instability and food insecurity, so as to holistically promote the well-being of the homeless.

### Limitations

This study has several limitations. First, the lack of sampling frame for recruitment may limit the representativeness of our findings to the entire homeless population in Hong Kong, despite a lack of reference demographic distribution of the local homeless community for valid comparisons. The age structure of our sample (i.e. mean age of 56·8) appears to be comparable with the official registry of sleep sleepers (i.e. around two-thirds aged 50 or above in those sleeping rough on the street) in Hong Kong^(6)^ whereas the small share of homeless women (i.e. 16·1 %) is also similar to the OECD statistics on homelessness (i.e. 22 % on average based on flow data^(53)^). Nonetheless, unlike most other studies, the similar proportion of ethnic minorities between our sample (i.e. 8·7 %) and the general Hong Kong population (i.e. 8·4 %^(54)^) suggests that the housing unaffordability issue may be equally affecting the socially disadvantaged groups regardless of ethnicity in Hong Kong. Also, as data collection was performed from evening to late night, homeless people who were working or highly mobile at night may be under-represented in our sample despite effort by experienced social workers to maximise the reach via careful planning of the routes and the most populated spots of homeless people. Second, given the cross-sectional design of this study, no temporal sequence and casual inference of our findings could be established. Also, the dynamic nature of homelessness and possible housing transitions of our recruited homeless individuals could not be captured in this study. Third, data collection via surveys relied on self-reported measures, which may be prone to recall error and social desirability bias. Fourth, there were a substantial proportion of missing data under highly constrained settings for data collection, although multiple imputation by chained equations were performed so that the regression analysis was conducted with the complete imputed dataset. Fifth, due to time concerns, the survey could not capture the full range of determinants of food insecurity. Other potential predictors, such as the frequency of receiving food support, entitlement to allowances other than CSSA, health literacy level and additional details related to homeless history and experiences, should be considered in future studies. Last, our findings regarding the at-risk groups and predictors were specific to the risk of food insecurity within the context of homelessness and should not be generalised to other risks and vulnerabilities, at personal, interpersonal or structural levels, that the homeless community may also face.

### Conclusion

Food insecurity is prevalent and remains a priority concern among the homeless population in the highly developed setting of Hong Kong. Nonetheless, there is substantial heterogeneity within the homeless population, suggesting the complexity and diversity of food access patterns shaped by the unique balance between resources and obstacles across individuals. Our findings underscore that conventional socio-demographic indicators of vulnerability in the general population may not necessarily align with obstacles to food access within the homeless community. Addressing the multifaceted nature of homeless experiences and its intersections with food insecurity requires targeted support for mobile and dispersed homeless individuals as well as those facing frequent evictions and relocations. Moreover, considering the robust dose-response relationship between health status and food insecurity, interventions should prioritise homeless individuals with compromised physical and mental health. Moving forward, a comprehensive approach integrating health assessments into initiatives addressing housing instability and food insecurity is crucial to foster the well-being of the homeless population in Hong Kong.
